# Efficacy and Safety of Biosimilar Romiplostim Versus Innovator Romiplostim in Patients with Chronic Immune Thrombocytopenia

**DOI:** 10.1007/s12288-022-01602-5

**Published:** 2022-11-15

**Authors:** S. Chandrakala, Manoj Toshniwal, Mitesh Halvawala, Namita Padwal, Neeraj Sidharthan, Pankaj Malhotra, B. Prashantha, Riya Ballikar, Sandip Shah, Shashikant Apte, T. Kasi Viswanathan, Vijay Ramanan, Akhilesh Sharma, Dattatray Pawar, Roshan Pawar, Vinayaka Shahavi

**Affiliations:** 1 Seth G. S. Medical College and KEM Hospital, Mumbai, India; 2 Ishwar Institute of Health Care, Aurangabad, India; 3 Nirmal Hospital Pvt Ltd, Surat, India; 4 LTMMC and General Hospital, Sion, India; 5 Amrita Institute of Medical Sciences, Kochi, India; 6 Postgraduate Institute of Medical Education and Research, Chandigarh, India; 7 Kasturba Medical College (KMC) Hospital, Mangalore, India; 8 Criticare Hospital and Research Institute, Nagpur, India; 9 Hemato-Oncology Clinic, Ahmedabad, India; 10 Sahyadri Super Specialty Hospital, Pune, India; 11 Meenakshi Mission Hospital and Research Centre, Madurai, India; 12 Grant Medical Foundation Ruby Hall Clinic, Pune, India; 13 Medical Affairs Department, Alkem Laboratories Limited, Alkem House, Senapati Bapat Marg,Lower Parel, Mumbai, India

**Keywords:** Immune thrombocytopenia, Biosimilar romiplostim, Efficacy endpoint, Adverse event

## Abstract

Romiplostim is a Food and Drug Administration (FDA)-approved therapy for immune thrombocytopenia (ITP). Biosimilar is a biological product that has no clinical meaningful difference from an existing FDA-approved reference product. It has a potential of lowering health-care-related cost. Biosimilar of romiplostim can be made available to patients with ITP at a low cost and can be beneficial in providing the best therapy. Thus, the efficacy and safety of biosimilar romiplostim (ENZ110) was compared with innovator romiplostim (Nplate) with respect to platelet response in patients with chronic ITP. This was a prospective, multicenter, randomized, and double-blind clinical trial. Patients with chronic ITP, aged 18–65 years, were enrolled in a study and were randomized to receive either ENZ110 or Nplate in a 3:1 ratio for a treatment period of 12 weeks, respectively. After completion of the treatment period, the patients were followed-up for one week to evaluate the platelet response and to monitor the adverse events (AEs). Over the duration of 12 weeks, platelet response of > 50 × 10^9^/L was achieved in 85.3% patients treated with ENZ110 and in 75.0% patients treated with Nplate in per protocol population. In intent-to-treat population, 83.8% patients with ENZ110 and 76.9% patients with Nplate achieved a platelet response of > 50 × 10^9^/L. In the ENZ110 group, 111 AEs were recorded in 66.7% patients, while 18 AEs were reported in 61.5% patients in the Nplate group. The study demonstrated non-inferiority with comparable efficacy and safety between biosimilar romiplostim and innovator romiplostim in patients with chronic ITP.

*Trial registration number and date of registration*: CTRI/2019/04/018614.

## Introduction

Immune thrombocytopenia (ITP) is a prevalent hematologic disorder that affects people of all ages, genders, and ethnicities [1]. Idiopathic ITP is a condition of having a low platelet count (thrombocytopenia) of no known cause (idiopathic) [2]. Chronic ITP is described as a disease that lasts for more than 12 months [1]. Adults are most commonly affected by chronic ITP. These individuals require treatment since the condition seldom cures independently, and there is a risk of severe consequences [3].

Adult patients with ITP have higher rates of morbidity and mortality than the general population, especially those who cannot maintain a hemostatic platelet count > 30 × 10^9^/L despite treatment [4, 5]. The objective of therapy is to produce a hemostatic platelet count of at least 20–30 × 10^9^/L while producing the least amount of harm possible [4]. Current treatments aim to increase platelet counts in ITP patients, mainly by reducing platelet destruction [6].

First-line treatment includes IV immunoglobulin, steroids, anti-D-immunoglobulin, and lastly, splenectomy. Rituximab is an alternative treatment in patients who are at increased risk of bleeding after the failure of above treatments. Recombinant versions of human thrombopoietin (TPO) were the first generation of thrombopoietic agents [7]. TPO is the endogenous ligand for the TPO receptor, expressed on the surface of platelets and megakaryocytes, and is the key cytokine involved in thrombopoiesis [8, 9]. Because neutralizing autoantibodies cross-react with endogenous TPO, the development of these agents was stopped. As a result, second-generation thrombopoiesis-stimulating compounds with no sequence similarity to endogenous TPO were developed [8]. Romiplostim (ROM) and eltrombopag (ELT) has been authorized by the Food and Drug Administration (FDA) in 2008, European Medicines Agency (EMA), and the UK's Medicines and Healthcare Products Regulatory Agency (MHRA) for the treatment of primary ITP in adult patients who are refractory to other treatments [e.g., corticosteroids, immunoglobulins (Igs)] Romiplostim and eltrombopag have no sequence homology with TPO, thus decreasing the risk of antibody formation. [10–12].

Romiplostim is a peptibody or Fc-peptide fusion protein. It is made up of two identical single-chain subunits, each with two human IgG1 Fc domains covalently bonded at the C-terminus to a peptide with two TPO receptors (c-Mpl)–binding domains (four total binding sites) [13, 14]. Romiplostim is a recombinant DNA product made in *Escherichia coli* (*E. coli*) that resembles human TPO. By attaching to the TPO receptor, romiplostim activates intracellular transcriptional pathways, resulting in enhanced platelet synthesis. TPO receptor binding stimulates the development of bone marrow megakaryocyte colony-forming cells, resulting in enhanced platelet synthesis via the Janus kinase 2 (JAK2) and signal transducers and activators of transcription 5 (STAT5) kinase pathways [3].

ROM is a subcutaneously administered peptide mimetic binding to the extracellular TPO-receptor, while ELT is an oral non-peptide binding to a transmembrane site of the TPO-receptor. An indirect comparative study between two concluded that romiplostim significantly improved overall platelet response compared with eltrombopag, however the durable platelet response of the two was similar. Another indirect study concluded that overall response, the incidence of adverse events, durable response, the incidence of overall bleeding and clinically significant bleeding, and the proportion of patients receiving rescue treatment were similar between eltrombopag and romiplostim. However, studies had concluded that ELT is more cost effective than romiplostim [15].

A biosimilar is a biological product similar to an approved and marketed biological product known as the reference product. It has no clinically meaningful differences in terms of safety and effectiveness from the reference product. Biosimilars are believed to have a positive impact on drug pricing. Health-care experts and physicians are optimistic that the use of biosimilars will lower the cost of biologics and, as a result, improve patient's access to these life-saving drugs. The biosimilar of Romiplostim, ENZ110, would accomplish the unmet need in the niche Indian market in patients with ITP, its introduction would be an effective treatment option due to its affordability [16].

In the present study, we compared the efficacy and safety of biosimilar romiplostim i.e., ENZ110 with innovator romiplostim i.e., Nplate in terms of platelet response in patients with chronic ITP.

## Materials and Methods

### Study Design and Population

Male and female patients diagnosed with chronic ITP, aged 18–65 years were enrolled in a prospective, multicenter, randomized, double-blind clinical trial. After signing the informed consent form, these patients were selected. Subjects diagnosed with idiopathic ITP according to the American Society of Hematology guidelines, with a bone marrow biopsy report consistent with an ITP diagnosis if over 60 years old, having received at least one prior therapy for ITP, having a single platelet count of ≤ 30 × 10^9^/L at any time during the screening period, had splenectomy/non-splenectomy and willingly and ably providing written informed consent were included in the study.

Subgroup analysis was not done & was not considered while calculating sample size. This phase III study was conducted as per the regulatory requirements for marketing authorization to address the pre-market regulatory requirements including comparability exercise for quality, preclinical and clinical studies. The comparative Pharmacodynamic, Pharmacokinetic, Immunogenicity of the product and clinical trials are critical to demonstrate the similarity in safety and efficacy profiles between the Similar Biologic and Reference Biologic for the manufacturing and marketing authorization approval. The study was conducted as per the principles and requirements of Declaration of Helsinki, and International Conference on Harmonization-Good Clinical Practice (ICH-GCP) guidelines along with the local regulatory requirements of Good Clinical Practices for Clinical Research in India (2004, CDSCO), Indian Council of Medical Research (ICMR) guidelines for Biomedical Research on Human Subjects (2017), and New Drugs and Clinical Trial Rules, 2019 (CDSCO).

The study protocol was approved by the independent ethics committee at all the participating 14 Centre before any patient enrollment in the study at that site. Written informed consent was obtained from the patient before the patient underwent any protocol-specific screening or study procedures. The trial was registered with Clinical Trials Registry-India (CTRI).

Subjects with a history of haematological malignancy, myeloproliferative disorder, myelodysplastic syndrome (MDS), bone marrow stem cell disorder, congenital thrombocytopenia, systemic lupus erythematosus, Evans syndrome, autoimmune neutropenia, antiphospholipid antibody syndrome, disseminated intravascular coagulation, haemolytic uremic syndrome, thrombotic thrombocytopenic purpura, infection with *Helicobacter pylori*, chronic liver disease (Child–Pugh score ≥ 7), any thromboembolic disease or were known to be positive for lupus anticoagulant, or positive for hepatitis B, hepatitis C, or human immunodeficiency virus at screening were excluded from the study. Also, subjects with previous use of romiplostim, pegylated recombinant human megakaryocyte growth and development factor, Eltrombopag, recombinant human TPO, or any platelet producing agent, or having known hypersensitivity to any recombinant *E. coli*-derived product, or of reproductive potential and was not using adequate contraceptive precautions, in the judgment of the investigator, or was pregnant or breastfeeding, or was unable to comply with the protocol procedures, were excluded from the study.

### Treatment Plan

The study consisted of a screening period (up to one week), a treatment period (12 cycles-each of 7 days), and a follow-up period (one week after cycle 12). Upon fulfilment of the selection criteria, subjects were randomized to receive either biosimilar romiplostim or innovator romiplostim in a 3:1 ratio to enter into the treatment period of 12 weeks, respectively. During the treatment period, romiplostim (ENZ110 or Nplate) was administered subcutaneously to all the eligible patients once a week.

Romiplostim dose during the study week was adjusted according to the protocols. Suppose the platelet count was < 50 × 10^9^/L, the dose was increased by 1 mcg/kg, if platelet count was > 200 × 10^9^/L for two consecutive weeks, the dose was reduced by 1 mcg/kg, if platelet count was > 400 × 10^9^/L, no dose was administered, and platelet count was assessed weekly, and if platelet count decreased to < 200 × 10^9^/L, romiplostim was resumed at a dose reduced by 1 mcg/kg against the last received dose. The weekly dose did not exceed more than 10 mcg/kg. Responders were defined as patients achieving platelet count ≥ 50 × 10^9^/L, i.e., sufficient platelet count to avoid clinically significant bleeding at the maximum weekly dose of 10 mcg/kg.

Rescue medications were permitted at the investigator's discretion, or when the subjects experienced bleeding, wet purpura, were at immediate risk for haemorrhage, or when the platelet count did not increase to a level sufficient to avoid clinically significant bleeding at the maximum weekly dose of 10 mcg/kg. Rescue medications administered were corticosteroid, intravenous immune globulin, anti-D Ig, and platelet transfusions.

### Study Assessments

After completion of the treatment period, the patients were followed up for one week to assess platelet response and monitor the adverse events (AEs). During the clinical trial, complete blood count (CBC), including peripheral blood smear (PBS), platelet count, prothrombin time/international normalized ratio (PT/INR), activated partial thromboplastin time (aPTT), were evaluated every week for ensuring patient safety as it not only play a role in coagulation but also in host defence against infection, and aspartate aminotransferase (AST), alanine transaminase (ALT), alkaline phosphatase (ALP), bilirubin, and serum creatinine were assessed every 4 weeks before the dose of romiplostim.

For the pharmacokinetic (PK) sub-study, blood samples of volume 5 mL in BD vacutainer were collected pre-injection within 30 min before the start of romiplostim injection (0 min) at cycle 1 and post-injection at 1 h (± 5 min), 2 h (± 5 min), 4 h (± 5 min), 6 h (± 5 min), 12 h (± 5 min), 15 h (± 5 min), 18 h (± 5 min), 21 h (± 5 min), 24 h (± 1 h), 48 h (± 1 h), and 72 h (± 1 h). Biological assay was done with standard Enzyme linked Immunosorbent assays (ELISA) method at Enzene bioscience limited.

Anti-romiplostim antibodies were assessed across both the treatment groups. Blood samples were collected before the start of the romiplostim administration at cycle one and at the end of study for efficacy endpoints and after week 24 (post-study for immunogenicity assessment).

### Efficacy Endpoints

The primary endpoint included a proportion of patients achieving platelet response (achievement of a weekly platelet count ≥ 50 × 10^9^/L) in both the study groups, and secondary endpoints were single-dose truncated PK parameters (C_max_, T_max_, AUC_0-t_) and incidence of treatment-emergent AEs (TEAEs) in both the treatment groups during the study period, and presence of anti-romiplostim antibody at baseline, end of study (EOS) visit, and after week 24 (post-study) visit.

### Statistical Analysis

A formal non-inferiority test was conducted for the primary endpoint. The null and alternative hypotheses for non-inferiority testing are given below:

*H0: p[test]—p[reference]* > *Δ*

*HA: p[test]—p[reference]* < *Δ*

H0 is the null hypothesis whereas HA is the alternative hypothesis, Δ is the margin of non-inferiority, which is already defined in the protocol (i.e., 20%), *p*[test]: proportion of patients achieving platelet response in the test group, *p*[reference]: proportion of patients achieving platelet response in the reference group.

A response rate was defined as the proportion of patients achieving a weekly platelet count ≥ 50 × 10^9^/L within 12 weeks of romiplostim treatment in both the study groups. The chi-square test was used for the comparison of two proportions from the two treatment groups. In addition to a *p*-value of the test, two-sided 90% confidence interval (CI) for the difference were calculated. If the lower limit of the 90% CI is more significant than zero, the proportion was estimated with sufficient precision. Proportion of patients with platelet count ≥ 50 × 10^9^/L for six or more times during the last 8 weeks of treatment study in both the study groups were summarized using counts (N: number of subjects per treatment group, n: number of subjects with non-missing values) and percentages.

## Results

### Patient Population and Demographic

**Fig. 1:**
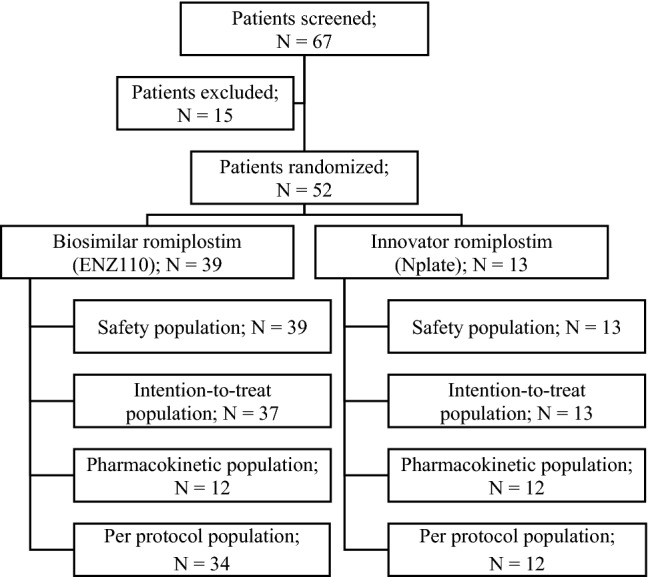
Patient disposition

In this multicentre study, a total of 67 subjects were screened from 14 clinical sites in India, and 52 were randomized (39 in biosimilar romiplostim arm [ENZ110], 13 in innovator romiplostim arm [Nplate]). Out of these, 46 (88.5%) patients [ENZ110, 34 (87.2%); Nplate, 12 (92.3%)] completed the study. The patient disposition is described in Fig. 1. No significant differences in the baseline characteristics were observed between the two treatment groups (Table 1).

**Table 1:** Baseline demographic characteristics of patients

Baseline characteristics	ENZ110	Nplate	Overall
	(n = 39)	(n = 13)	(n = 52)
Age (years), mean (SD)	37.97 (13.19)	33.54 (10.81)	36.87 (12.68)
Height (cm), mean (SD)	157.75 (9.53)	157.65 (3.80)	157.72 (8.43)
Weight (kg), mean (SD)	63.76 (13.94)	60.93 (12.01)	63.05 (13.43)
*Gender*			
Female, n (%)	25 (64.1)	10 (76.9)	35 (67.3)
Male, n (%)	14 (35.9)	3 (23.1)	17 (32.7)

*SD* standard deviation, *ENZ110* biosimilar romiplostim, *Nplate* innovator romiplostim

### Efficacy Analysis

#### Summary of Proportion of Patients Achieving a Weekly Platelet Count (Per Protocol [PP] and Intention-to-Treat [ITT] Population)

**Fig. 2:**
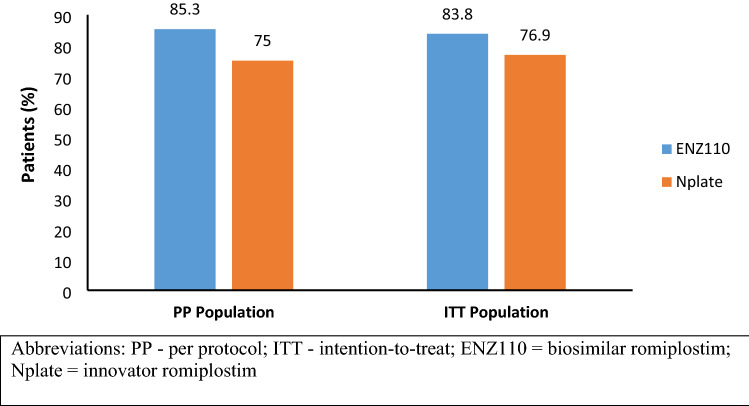
Proportion of patients responding to the treatment during the 12 weeks of romiplostim treatment. PP–per protocol; ITT–intention-to-treat; ENZ110 = biosimilar romiplostim; Nplate = innovator romiplostim

For the PP population, 46 patients were considered (34 patients in ENZ110 group; 12 patients in Nplate group); whereas for the ITT population, 50 patients were evaluated (37 patients in ENZ110 group; 13 patients in Nplate group) (Fig. 2). In over 12 weeks, a response of > 50 × 10^9^/L platelet count was achieved in 29 (85.3%) patients with ENZ110 and 9 (75.0%) patients with Nplate in the PP population and 31 (83.8%) patients with ENZ110 and 10 (76.9%) patients with Nplate in the ITT population (Fig. 2). Statistically significant response was noted in both the groups from baseline to week 12 in both the populations (*p* value for response in both groups was < 0.0001).

The other evaluation including CBC, AST, ALT were within normal range and showed no significant difference from baseline to the end of study.

### Pharmacokinetic and Immunogenicity Endpoints

The research enrolled a total of 24 individuals as planned. After subcutaneous injection of 0.1, 0.3, and 1.0 mcg/kg romiplostim, no measurable quantities were detected, according to the innovator's PK investigations. Only two out of eight individuals exhibited measurable amounts at dosage 2.0 mcg/kg. Based on this finding, a larger dosage of 3 mcg/kg subcutaneous was used in a later PK investigation in chronic ITP patients. Two patients in the ENZ110 group tested positive for anti-drug antibody (ADA), whereas one patient in the Nplate group tested positive.

### Safety Analysis

A total of 129 TEAEs were reported by 34 (65.4%) individuals (Table 2). Five serious AEs (SAEs) were reported out of which one SAE led to death (Table 3). Out of 129 TEAEs, 83 AEs were possibly related to the study medication, 4 AEs were probably related, and 42 AEs were unlikely to be related. In the ENZ110 group, 111 AEs were recorded in 26 (66.7%) patients, while 18 AEs were reported in 8 (61.5%) patients in the Nplate group. The proportion of patients experiencing at least one AE were similar between the groups.

**Table 2:** Summary of TEAEs

	ENZ110	Nplate	Overall
	(N = 39)	(N = 13)	(N = 52)
All TEAEs, n (%) E	26 (66.7) 111	8 (61.5) 18	34 (65.4) 129
*p*-value for difference in the incidence of AEs between treatment	0.8679		
TEAEs related to investigational product, n (%) E			
Possible	15 (38.5) 71	5 (38.5) 12	20 (38.5) 83
Probable/Likely	2 (5.1) 3	1 (7.7) 1	3 (5.8) 4
Unlikely	20 (51.3) 37	3 (23.1) 5	23 (44.2) 42
Severity of TEAEs, n (%) E			
Mild	21 (53.8) 76	8 (61.5) 16	29 (55.8) 92
Moderate	12 (30.8) 29	1 (7.7) 2	13 (25.0) 31
Severe	6 (15.4) 6	0 (0.0) 0	6 (11.5) 6
Seriousness of TEAEs, n (%) E			
Hospitalization or prolongation of hospitalization	4 (10.3) 4	0 (0.0) 0	4 (7.7) 4
Death	1 (2.6) 1	0 (0.0) 0	1 (1.9) 1
TEAEs leading to discontinuation of IMP	3 (7.7) 3	0 (0.0) 0	3 (5.8) 3

*E* number of events, *N* number of subjects dosed with each treatment, *n* number of subjects with adverse event with particular category, % calculated using the number of subjects treated with each treatment as the denominator (n/N)*100, *TEAEs* treatment-emergent adverse events, *ENZ110* biosimilar romiplostim, *Nplate* innovator romiplostim, *IMP* investigational medicinal product

**Table 3:** Summary of TEAEs reported by at least 5% subjects

System organ class	ENZ110	Nplate	Overall
Preferred term N (%) E	(N = 39)	(N = 13)	(N = 52)
*Blood And lymphatic system disorders*			
Anemia	3 (7.7) 3	1 (7.7) 1	4 (7.7) 4
*Cardiac disorders*			
Sinus tachycardia	0 (0.0) 0	1 (7.7) 2	1 (1.9) 2
*Gastrointestinal disorders*			
Abdominal pain upper	2 (5.1) 4	0 (0.0) 0	2 (3.8) 4
Constipation	2 (5.1) 3	0 (0.0) 0	2 (3.8) 3
Diarrhea	2 (5.1) 4	0 (0.0) 0	2 (3.8) 4
Dysphagia	0 (0.0) 0	1 (7.7) 1	1 (1.9) 1
Gastritis	2 (5.1) 4	0 (0.0) 0	2 (3.8) 4
Vomiting	2 (5.1) 2	0 (0.0) 0	2 (3.8) 2
*General disorders and administration site conditions*			
Chills	2 (5.1) 2	0 (0.0) 0	2 (3.8) 2
Non-cardiac chest pain	2 (5.1) 2	0 (0.0) 0	2 (3.8) 2
Pyrexia	6 (15.4) 6	1 (7.7) 1	7 (13.5) 7
*Infections and infestations*			
Pharyngitis	0 (0.0) 0	1 (7.7) 1	1 (1.9) 1
Upper respiratory tract infection	10 (25.6) 13	2 (15.4) 2	12 (23.1) 15
*Investigations*			
Liver function test abnormal	0 (0.0) 0	1 (7.7) 1	1 (1.9) 1
*Musculoskeletal and connective tissue disorders*			
Arthralgia	0 (0.0) 0	1 (7.7) 1	1 (1.9) 1
Muscle spasms	0 (0.0) 0	1 (7.7) 1	1 (1.9) 1
Musculoskeletal pain	0 (0.0) 0	1 (7.7) 1	1 (1.9) 1
Pain in extremity	3 (7.7) 4	1 (7.7) 1	4 (7.7) 5
Pain in jaw	2 (5.1) 2	0 (0.0) 0	2 (3.8) 2
*Nervous system disorders*			
Headache	5 (12.8) 7	0 (0.0) 0	5 (9.6) 7
*Renal And urinary disorders*			
Dysuria	0 (0.0) 0	1 (7.7) 1	1 (1.9) 1
*Reproductive system and breast disorders*			
Menorrhagia	3 (7.7) 3	0 (0.0) 0	3 (5.8) 3
*Skin and subcutaneous tissue disorders*			
Acne	2 (5.1) 2	0 (0.0) 0	2 (3.8) 2
Dry skin	2 (5.1) 2	0 (0.0) 0	2 (3.8) 2
Pruritus	4 (10.3) 5	1 (7.7) 2	2 (3.8) 2
*Vascular disorders*			
Ecchymosis	3 (7.7) 7	0 (0.0) 0	3 (5.8) 7
Petechiae	2 (5.1) 2	0 (0.0) 0	2 (3.8) 2
Vaginal hemorrhage	0 (0.0) 0	1 (7.7) 2	1 (1.9) 2

*E* number of events, *N* number of subjects dosed with each treatment, *n* number of subjects with adverse event with particular category, % calculated using the number of subjects treated with each treatment as the denominator (n/N)*100, *TEAEs* treatment-emergent adverse events, *ENZ110* biosimilar romiplostim, *Nplate* innovator romiplostim

## Discussion

The study conducted on ITP patients over a duration of 12 weeks showed non-inferiority between biosimilar romiplostim and innovator romiplostim. The platelet response of > 50 × 10^9^/L was achieved in 85.3% in patients treated with ENZ110 and 75% in patients treated with Nplate with no statistically significant difference in the incidence of TEAE between the two groups.

With respect to PK assessment, the majority of the samples were reported below the lower limit of quantitation (LLOQ) (40 pg/mL) of the assay. Hence, the statistical analysis was not performed.

No statistically significant difference observed in the incidence of TEAEs between the two treatment groups. These reported AEs were expected and consistent with reference to the romiplostim (Nplate) [12]. Treatment-emergent ADA was detected in two patients (5.71%) from ENZ110 group and one patient (9.09%) from Nplate group, which was consistent with the Summary of Product literature [12].

Romiplostim is an approved treatment in ITP. However, in India, due to cost constraints, the majority prefer immunosuppression therapy. The biosimilar of romiplostim, ENZ110, would come as a huge relief to patients with ITP, as its affordable cost would fulfill an unmet need in patients requiring the best treatment [17].

Two multicenter, placebo-controlled phase III trials were conducted simultaneously. These studies included 63 splenectomized and 62 non-splenectomized patients who had chronic ITP and a mean of three platelet counts of up to 30 × 10^9^/L. The overall platelet response rate was noted in 88% of non-splenectomized and 79% of splenectomized patients given romiplostim compared with 14% of non-splenectomized and no splenectomized patients given placebo (*p* < 0.0001) [18].

Another prospective, multicenter, randomized, double-blind study compared the efficacy and safety of biosimilar romiplostim with innovator romiplostim in patients with chronic ITP. Non-inferiority was statistically demonstrated for the primary efficacy endpoint between the biosimilar romiplostim and the innovator romiplostim. Proportion of patients achieving a weekly platelet count ≥ 50 × 10^9^/L was 85.3% with ENZ110 and 75.0% with Nplate over 12 weeks. The study achieved its non-inferiority efficacy endpoint as lower bound of the 90% two-sided CI (− 8.36, 28.94%) was greater than − 20%.

In a PK study conducted for innovator Nplate, maximum romiplostim serum levels in ITP patients were attained after 7–50 h following subcutaneous dose of 3–15 mcg/kg romiplostim (median 14 h). Patients' blood concentrations varied and were not related to the dose given and Romiplostim's elimination half-life in ITP patients ranged from 1 to 34 days. No accumulation in serum concentrations was observed after six weekly doses of 3 mcg/kg [10]. Since our study used 1 mcg/kg dose of ENZ110, there was no statistically significant difference observed in change from baseline to week 12 for hematology and biochemistry laboratory parameters in both the treatment groups.

## Conclusion

This study established non-inferiority, along with comparable safety and immunogenicity between biosimilar romiplostim and innovator romiplostim in patients with chronic ITP.

## References

[1] Kistangari G, McCrae KR (2013). Immune thrombocytopenia. Hematol Oncol Clin North Am.

[2] Kayal L, Jayachandran S, Singh K (2014). Idiopathic thrombocytopenic purpura. Contemp Clin Dent.

[3] Hubulashvili D, Marzella N (2009). Romiplostim (Nplate), a treatment option for immune (Idiopathic) thrombocytopenic purpura. P T.

[4] Portielje JE, Westendorp RG, Kluin-Nelemans HC, Brand A (2001). Morbidity and mortality in adults with idiopathic thrombocytopenic purpura. Blood.

[5] Nørgaard M, Jensen AØ, Engebjerg MC, Farkas DK, Thomsen RW, Cha S (2011). Long-term clinical outcomes of patients with primary chronic immune thrombocytopenia: a Danish population-based cohort study. Blood.

[6] Bussel JB, Kuter DJ, Pullarkat V, Lyons RM, Guo M, Nichol JL (2009). Safety and efficacy of long-term treatment with romiplostim in thrombocytopenic patients with chronic ITP. Blood.

[7] Kuter DJ (2007). New thrombopoietic growth factors. Blood.

[8] Stasi R, Bosworth J, Rhodes E, Shannon MS, Willis F, Gordon-Smith EC (2010). Thrombopoietic agents. Blood Rev.

[9] Kaushansky K (2005). The molecular mechanisms that control thrombopoiesis. J Clin Invest.

[10] Nplate prescribing information. 2011. https://www.accessdata.fda.gov/drugsatfda_docs/label/2011/125268s077lbl.pdf. Accessed 31 Oct 2021

[11] Nplate summary of product characteristics. 2020. https://www.ema.europa.eu/en/documents/product-information/nplate-epar-product-information_en.pdf. Accessed 31 Oct 2021

[12] Nplate 250 micrograms powder and solvent for solution for injection (Reconstitution Pack). 2021.https://www.medicines.org.uk/emc/product/567/smpc#gref. Accessed 24 Aug 2021

[13] Wang B, Nichol JL, Sullivan JT (2004). Pharmacodynamics and pharmacokinetics of AMG 531, a novel thrombopoietin receptor ligand. Clin Pharmacol Ther.

[14] Wang YM, Krzyzanski W, Doshi S, Xiao JJ, Pérez-Ruixo JJ, Chow AT (2010). Pharmacodynamics-mediated drug disposition (PDMDD) and precursor pool lifespan model for single dose of romiplostim in healthy subjects. AAPS J.

[15] Allen R, Bredyn P, Grotzinger K, Stapelkamph C (2016). Cost-effectiveness of eltrombopag versus romiplostim for the treatment of chronic immune thrombocytopenia in England and Wales. Value in Health.

[16] Meher BR, Balan S, Mohanty RR, Jena M, Das S (2019). Biosimilars in India; current status and future perspectives. J Pharm Bioallied Sci.

[17] Kuter DJ, Newland A, Chong BH, Rodeghiero F, Romero MT, Pabinger I (2019). Romiplostim in adult patients with newly diagnosed or persistent immune thrombocytopenia (ITP) for up to 1 year and in those with chronic ITP for more than 1 year: a subgroup analysis of integrated data from completed romiplostim studies. Br J Haematol.

[18] Bussel JB, Kuter DJ, George JN, McMillan R, Aledort LM, Conklin GT (2006). AMG 531, a thrombopoiesis-stimulating protein, for chronic ITP. N Engl J Med.

