# Correction to: Efficacy and Safety of Biosimilar Romiplostim Versus Innovator Romiplostim in Patients with Chronic Immune Thrombocytopenia

**DOI:** 10.1007/s12288-023-01635-4

**Published:** 2023-02-27

**Authors:** S. Chandrakala, Manoj Toshniwal, Mitesh Halvawala, Namita Padwal, Neeraj Sidharthan, Pankaj Malhotra, B. Prashantha, Riya Ballikar, Sandip Shah, Shashikant Apte, T. Kasi Viswanathan, Vijay Ramanan, Akhilesh Sharma, Dattatray Pawar, Roshan Pawar, Vinayaka Shahavi

**Affiliations:** 1 Seth G. S. Medical College and KEM Hospital, Mumbai, India; 2 Ishwar Institute of Health Care, Aurangabad, India; 3 Nirmal Hospital Pvt Ltd, Surat, India; 4 LTMMC and General Hospital, Sion, India; 5 Amrita Institute of Medical Sciences, Kochi, India; 6 Postgraduate Institute of Medical Education and Research, Chandigarh, India; 7 Kasturba Medical College (KMC) Hospital, Mangalore, India; 8 Criticare Hospital and Research Institute, Nagpur, India; 9 Hemato-Oncology Clinic, Ahmedabad, India; 10 Sahyadri Super Specialty Hospital, Pune, India; 11 Meenakshi Mission Hospital and Research Centre, Madurai, India; 12 Grant Medical Foundation Ruby Hall Clinic, Pune, India; 13 Medical Affairs Department, Alkem Laboratories Limited, Alkem House, Senapati Bapat Marg, Lower Parel, Mumbai, India

**Correction to: Indian J Hematol Blood Transfus** 10.1007/s12288-022-01602-5

The article “Efficacy and Safety of Biosimilar Romiplostim Versus Innovator Romiplostim in Patients with Chronic Immune Thrombocytopenia” written by S. Chandrakala, Manoj Toshniwal, Mitesh Halvawala, Namita Padwal, Neeraj Sidharthan, Pankaj Malhotra, B. Prashantha, Riya Ballikar, Sandip Shah, Shashikant Apte, T. Kasi Viswanathan, Vijay Ramanan, Akhilesh Sharma, Dattatray Pawar, Roshan Pawar and Vinayaka Shahavi was originally published without Open Access.

After online publication, the author decided to opt for Open Choice and to make the article an Open Access publication. Therefore, the copyright of the article has been changed to © The Author(s) 2022 and the article is forthwith distributed under the terms of the Creative Commons Attribution 4.0 International License, which permits use, sharing, adaptation, distribution and reproduction in any medium or format, as long as you give appropriate credit to the original author(s) and the source, provide a link to the Creative Commons licence, and indicate if changes were made. The images or other third party material in this article are included in the article’s Creative Commons licence, unless indicated otherwise in a credit line to the material. If material is not included in the article’s Creative Commons licence and your intended use is not permitted by statutory regulation or exceeds the permitted use, you will need to obtain permission directly from the copyright holder. To view a copy of this licence, visit http://creativecommons.org/licenses/by/4.0.

The original article has been corrected.

